# FGF1 supports glycolytic metabolism through the estrogen receptor in endocrine-resistant and obesity-associated breast cancer

**DOI:** 10.1186/s13058-023-01699-0

**Published:** 2023-08-22

**Authors:** Marisol Castillo-Castrejon, Barbara Mensah Sankofi, Stevi Johnson Murguia, Abasi-ama Udeme, Hoaning Howard Cen, Yi Han Xia, Nisha S. Thomas, William L. Berry, Kenneth L. Jones, Vincent R. Richard, Rene P. Zahedi, Christoph H. Borchers, James D. Johnson, Elizabeth A. Wellberg

**Affiliations:** 1 Department of Pathology, University of Oklahoma Health Sciences Center, 975 NE 10th Street BRC 309, Oklahoma City, OK 73104 USA; 2 Life Sciences Institute, University of British Columbia, Vancouver, Canada; 3 Segal Cancer Proteomics Centre, Lady Davis Institute, Jewish General Hospital and McGill University, Montreal, QC Canada; 4 Manitoba Centre for Proteomics and Systems Biology, Winnipeg, MB R3E 3P4 Canada; 5 Department of Internal Medicine, University of Manitoba, Winnipeg, MB R3E 3P4 Canada; 6 Department of Biochemistry and Medical Genetics, University of Manitoba, Winnipeg, MB R3E 0J9 Canada; 7 CancerCare Manitoba Research Institute, Winnipeg, MB R3E 0V9 Canada; 8 Gerald Bronfman Department of Oncology, Lady Davis Institute for Medical Research, Jewish General Hospital, Montreal, QC H3T 1E2 Canada; 9 Division of Experimental Medicine, McGill University, Montreal, QC H4A 3J1 Canada; 10 Department of Pathology, McGill University, Montreal, QC H3A 2B4 Canada

**Keywords:** Breast cancer, Adipose, Obesity, Estrogen receptor, Fibroblast growth factor

## Abstract

**Background:**

Obesity increases breast cancer risk and breast cancer-specific mortality, particularly for people with estrogen receptor (ER)-positive tumors. Body mass index (BMI) is used to define obesity, but it may not be the best predictor of breast cancer risk or prognosis on an individual level. Adult weight gain is an independent indicator of breast cancer risk. Our previous work described a murine model of obesity, ER-positive breast cancer, and weight gain and identified fibroblast growth factor receptor (FGFR) as a potential driver of tumor progression. During adipose tissue expansion, the FGF1 ligand is produced by hypertrophic adipocytes as a stimulus to stromal preadipocytes that proliferate and differentiate to provide additional lipid storage capacity. In breast adipose tissue, FGF1 production may stimulate cancer cell proliferation and tumor progression.

**Methods:**

We explored the effects of FGF1 on ER-positive endocrine-sensitive and resistant breast cancer and compared that to the effects of the canonical ER ligand, estradiol. We used untargeted proteomics, specific immunoblot assays, gene expression profiling, and functional metabolic assessments of breast cancer cells. The results were validated in tumors from obese mice and breast cancer datasets from women with obesity.

**Results:**

FGF1 stimulated ER phosphorylation independently of estradiol in cells that grow in obese female mice after estrogen deprivation treatment. Phospho- and total proteomic, genomic, and functional analyses of endocrine-sensitive and resistant breast cancer cells show that FGF1 promoted a cellular phenotype characterized by glycolytic metabolism. In endocrine-sensitive but not endocrine-resistant breast cancer cells, mitochondrial metabolism was also regulated by FGF1. Comparison of gene expression profiles indicated that tumors from women with obesity shared hallmarks with endocrine-resistant breast cancer cells.

**Conclusions:**

Collectively, our data suggest that one mechanism by which obesity and weight gain promote breast cancer progression is through estrogen-independent ER activation and cancer cell metabolic reprogramming, partly driven by FGF/FGFR. The first-line treatment for many patients with ER-positive breast cancer is inhibition of estrogen synthesis using aromatase inhibitors. In women with obesity who are experiencing weight gain, locally produced FGF1 may activate ER to promote cancer cell metabolic reprogramming and tumor progression independently of estrogen.

**Supplementary Information:**

The online version contains supplementary material available at 10.1186/s13058-023-01699-0.

## Introduction

Obesity is an established risk factor for breast cancer development and breast cancer-specific mortality [1–5]. BMI, which defines obesity, allows population-level analyses of disease risks, but may not be the best way to evaluate an individual’s risk for specific diseases, including breast cancer [6]. Adult weight gain has emerged as a potentially strong predictor of breast cancer risk, irrespective of BMI [7, 8]. During weight gain, adipose tissue expands and produces numerous growth factors. Among these is fibroblast growth factor 1 (FGF1), which we previously showed is elevated in mammary adipose of obese female mice during weight gain that occurs after ovariectomy and which is directly correlated with BMI in breast adipose tissue from women [9]. FGF1 is produced by hypertrophic adipocytes and stimulates proliferation of adipose stromal cells to promote tissue expansion characterized by hyperplasia and de novo adipogenesis [10]. The FGF1-stimulated hyperplastic growth of adipose tissue during a positive energy balance is beneficial to the individual, as it prevents lipodystrophy and ectopic lipid deposition. However, growth factors produced by adipocytes can also stimulate nearby cancer cells, particularly in the breast where adipose and epithelium are in proximity to each other [6].

Our prior studies demonstrated that some, but not all, estrogen receptor-positive (ER-positive) tumors progressed in the obese environment after ovariectomy (OVX) and withdrawal of supplemental estradiol [9]. Those that did progress in obesity had elevated levels of phosphorylated FGFR1 and pharmacological inhibition of FGFR signaling restored sensitivity to estrogen deprivation. In human specimens, phosphorylated FGFR1 in tumors was associated with a shorter breast cancer-specific survival after tamoxifen treatment and was also elevated in tumors from patients with a high BMI [9]. The purpose of this study was to understand the distinct effects of FGF1 exposure in ER-positive breast cancers that show sensitivity or resistance to estrogen deprivation in the context of obesity.

FGFRs are among several growth factor receptors that can crosstalk with steroid hormone receptors and promote their activation independently of the canonical hormone ligands [9, 11–13]. FGFR1 is amplified in up to 15% of luminal breast tumors and has been shown to translocate to the nucleus of breast cancer cells, where it stimulates target gene expression [11]. FGFR activation is associated with and permits endocrine therapy resistance in ER-positive breast tumors [13, 14]. Unfortunately, therapies targeting FGFR signaling have had mixed results in clinical trials, potentially due to inaccurate patient selection, overestimation of the number of patients with FGFR-dependent tumors, or inappropriate dosing and toxicity [15]. One strategy to improve the efficacy of FGF/FGFR-targeted therapies may involve a better understanding of the downstream effects of these signaling pathways in different tumors, which may further refine patient selection based on specific tumor characteristics beyond DNA alterations. Here, we show that FGF1 can stimulate ER phosphorylation and activation in some breast cancer cells and we suggest that one important effect is metabolic reprogramming of aggressive cancer cells toward a glycolytic phenotype.

## Methods

### Mouse studies

Female Rag1KO mice were purchased from Jackson Laboratories at 6 weeks of age (B6.129S7-Rag^1tm1Mom^/J; stock #002216). Mice were housed on warming blankets and given a high-fat (40%) or low-fat (11%) diet as previously described [9, 16]. Diets were purchased from Research Diets, Inc. At approximately 18 weeks of age, mice were ovariectomized and immediately supplemented with 17β-estradiol administered in drinking water (E2; final 0.5 μM). Tumor cells (MCF7 or MCF7 TAMR) or 2 mm × 2 mm fragments (UCD12) were implanted in the inguinal mammary fat pads. When tumors reached 500–700 mm^3^, supplemental E2 was withdrawn, and the study was ended 3 weeks later. Tumors were measured weekly with digital calipers. Mice were fasted for 4 h prior to killing. Body composition was determined with qMR (ECHO MRI).

### Cell lines and reagents

MCF7 and tamoxifen-resistant MCF7 (TAMR) cells were purchased from ATCC and were maintained as suggested by the vendor in complete growth media. UCD12 cells were developed by the University of Colorado Denver/Anschutz Medical Campus, as described [17]. During experimental treatments, cells were starved for 16 h in phenol red-free Dulbecco’s modified Eagle medium (DMEM) containing 0.5% charcoal-stripped fetal bovine serum to reduce the potential for steroid receptor activation. All hormone or growth factor treatments were given in this media. 17β-Estradiol was purchased from Sigma-Aldrich, resuspended in EtOH vehicle, and used at a final concentration of 10 nM. Fulvestrant (ICI182780) was purchased from Sigma-Aldrich, resuspended in DMSO, and used at a final concentration of 100 nM. Recombinant human FGF1 was purchased from R&D Systems, resuspended in PBS vehicle, and used at a final concentration of 5 ng/mL.

To generate FGFR1-overexpressing MCF7 cells, plasmids pHAGE BFP (Addgene #106282) and pHAGE FGFR1 (Addgene #116740) were transfected into HEK 293 T cells along with psPax2 (Addgene #12260) and pMD2.G (Addgene #12259) using Transporter 5 (Polysciences). Cell supernatant was collected at 48 h and 72 h post-transfection and then filtered with a 0.45-micron PVDF filter. Sterile polyethylene glycol in water was added to a final concentration of 8%. PEG–virus solution was centrifuged for 30 min at 1500xg. Supernatant was discarded and virus was resuspended in Opti-MEM I (Thermo Fisher). MCF7 cells were transduced in the presence of polybrene (10 µg/mL), and a polyclonal population of cells was generated by using puromycin (1 µg/mL) to kill untransduced cells.

### Proteomics and phospho-proteomics

For the proteomics analyses, normalization and imputation were performed on the protein abundances using Proteome Discoverer 2.5 (Thermo Scientific). We plotted the log_2_ (fold change) of the tamoxifen-resistant MCF7 control over the parental MCF7 control. Differentially abundant proteins (abs(log_2_(fold change)) > 5) between these two groups were used as input into STRING [18] to create protein–protein interaction diagrams using default settings. The network was visualized using Cytoscape (v3.9.1) [19], with background images created with BioRender.com. Proteins were placed based on their known intracellular organelle locations and functions or using the COMPARTMENTS section of GeneCards [20].

For the phospho-proteomics analyses, imputation was performed on the abundances using Proteome Discoverer. These were aggregated by sum for each repeated phospho-site and then normalized to the corresponding total protein data for each sample and protein. A heatmap was created using phospho-sites that were differentially abundant (abs(log_2_(fold change)) > 5) in any of the four comparisons (E2 vs control and FGF1 vs control for parental MCF7 and TAMR cells). The heatmap used k-means clustering, and clusters with expression patterns of interest were extracted [21]. The corresponding proteins from these phospho-site clusters were used as input into STRING [18] to create protein–protein interaction diagrams using default settings. The network was visualized using Cytoscape (v3.9.1) [19], with background images created with BioRender.com. Proteins were placed based on their known intracellular organelle locations and functions or using the COMPARTMENTS section of GeneCards [20].

### Capillary immunoblot analyses

Cells were harvested using a cell scraper after adding radioimmunoprecipitation (RIPA) buffer (Thermo Fisher Scientific, Rockford, IL) containing phosphatase inhibitor cocktail (Roche Cat. No 04906845001) and protease inhibitor cocktail (Roche Cat. No. 04693116001). Protein concentrations were determined by Pierce bicinchoninic acid (BCA) Protein Assay Kit (Thermo Fisher Scientific, Rockford, IL) and were stored at -80C until further processing. We evaluated the total protein and phosphorylated proteins in cell lysates by the Simple Western system that uses an automated capillary electrophoresis to perform protein separation (Protein Simple, San Jose, CA, SM-W004-1, PS-ST01, PN-009-050), immobilized the separated protein onto the capillary wall, immuno-probe for the target protein using a primary antibodies total ERα (Thermo Scientific RM9101-50), ERα S118 (Abcam Cat. No. 32396 diluted 1:50), ERα S167 (Cell Signaling Technologies, Boston, MA, Cat. No. 64508 s), p44/42 MAPK (Erk1/2)(Thr202/Thr204) (Cell Signaling Technologies, Boston, MA, Cat. No. 4370S diluted 1:200), p44/42 MAPK(Erk1/2) (Cell Signaling Technologies, Boston, MA, Cat. No. 9102 diluted 1:200) and vinculin was used as loading control (Cell Signaling Technologies, Boston, MA, 1390, diluted 1:1000) and secondary anti-rabbit HRP antibody conjugate (1X) (Protein Simple, San Jose, CA, DM-001). Washes were performed between antibody addition, and the signal was developed, identified, and quantified automatically. To facilitate comparisons, the individual values were normalized to the average of the mean levels for vehicle-treated cells in each experiment and expressed as fold change relative to vehicle.

### Metabolic flux analyses

Agilent Seahorse ATP rate assay was used to quantify the rate of ATP production from glycolysis and mitochondria respiration according to manufacturer’s instruction using the Seahorse XFe96 analyzer. Briefly, cells were seeded into seahorse XFe96 microplates at a density of 7.5 × 103 cells/well followed by treatment with EtOH (vehicle), 17β-estradiol (E2; 10 nM), 5 ng/ml recombinant FGF1 and in the presence or absence of fulvestrant in hormone-free media for 24 h. The sensor cartridges were hydrated in Seahorse XF calibrant at 37 °C in a non-CO2 incubator overnight. Next, the culture media was substituted with Seahorse XF DMEM medium supplemented with 1 mM of sodium pyruvate, 2 nM of glutamine, and 1 mM of d-glucose. The cells were placed in a CO2 incubator at 37 °C for 1 h prior to test. Cells were stimulated following the injection of 1.5 μM oligomycin and 0.5 μM rotenone/actimycin A to measure oxygen consumption (OCR) and extracellular acidification rate (ECAR). Total protein content was determined by Pierce BCA protein assay according to manufacturer’s instructions.

### RNA sequencing and bioinformatics

Cell lines were treated with 10 nM E2 or recombinant human FGF1 (5 ng/mL) for 24 h in the presence or absence of fulvestrant (ICI 182 780; 100 nM) following an overnight starvation period in phenol red-free media containing 0.5% charcoal-stripped fetal bovine serum. RNA was collected using the RNEasy mini kit (Qiagen). Total RNA was provided to the Genomics Core at the University of Oklahoma Health Sciences Center Institutional Research Core Facility and used to prepare mRNA libraries to be sequenced using 2 × 150 bp reads on an Illumina NovaSeq 6000. Sequences were then mapped to the human genome (GRCh38) by gSNAP [22] and expression (FPKM) derived by Cufflinks [23]. From that, differential expression was analyzed with ANOVA in R. Genes significant at a FDR < 0.05 were utilized for further downstream analysis. Kaplan–Meier curves for human breast cancer samples were generated using the KM Plotter database [24]. Gene set analysis of expression data was performed using the GSEA Molecular Signatures Database [25]. Venn diagrams were made using BioVenn [26]. Heatmaps were plotted with the hierarchical clustering of gene expressions (FPKM) using ComplexHeatmap R package [21]. Sequencing data are deposited in the NCBI Gene Expression Omnibus under GSE237784.

### Human dataset analyses

Raw data were obtained from the NCI Gene Expression Omnibus from GSE78958 [27] and GSE24185 [28]. The raw CEL files were processing using latest CDF map from http://brainarray.mbni.med.umich.edu/Brainarray/Database/CustomCDF/CDF_download.asp (version 25, GENECODET, HGU133A2 chip) and normalized using Iterative rank-order normalization (IRON) method [29]. Analyses were restricted to tumors that were ER-positive, HER2-negative (GSE24185; *N* = 10 lean, *N* = 14 obese), or luminal A/B (GSE78958; *N* = 81 lean, *N* = 96 obese). BatchQC package was used for quality check, which identified and removed nine outliers out of 182 samples in GSE78958, based on low pairwise sample correlations. In each case, tumors from the obese category (BMI ≥ 30) were compared to the lean/normal category (BMI < 25). The log_2_ fold changes and *p*-values of the gene expressions were determined by limma R package [30]. Gene set enrichment analysis (GSEA) was performed with rank matric score (− log_10_ *p*-value * sign of log_2_ fold change) in clusterProfiler R package [31] using the Hallmark gene sets from Molecular Signatures Database [25, 32]. Code is available at https://github.com/hcen/breastcancer_LW2023.

### Cell proliferation and BCA assays

Cells were seeded in complete growth media (200,000 cells/well) and allowed to adhere. The next day, cells were starved for 18 h in phenol red-free DMEM containing 0.5% charcoal-stripped fetal bovine serum. After starvation, cells were treated with either EtOH vehicle, 10 nM of E2, or 5 ng/mL of FGF1 in the presence or absence of fulvestrant (ICI 182 780) in hormone-free media for 24 h. Cells were trypsinized and counted using trypan blue each day for 3 days to assess the effect of each treatment on cell proliferation.

### Statistics

Statistical analyses were performed in Prism 9.4.1 or using the statistical programming language, R. Where appropriate, tests included ANOVA (2 × 2 with interaction test or one-way ANOVA) or *t*-tests. A p value of less than 0.05 was considered significant (Additional file 1).

## Results

### Proteomic and phospho-proteomic comparison of MCF7 and tamoxifen-resistant MCF7 cells

**Fig. 1:**
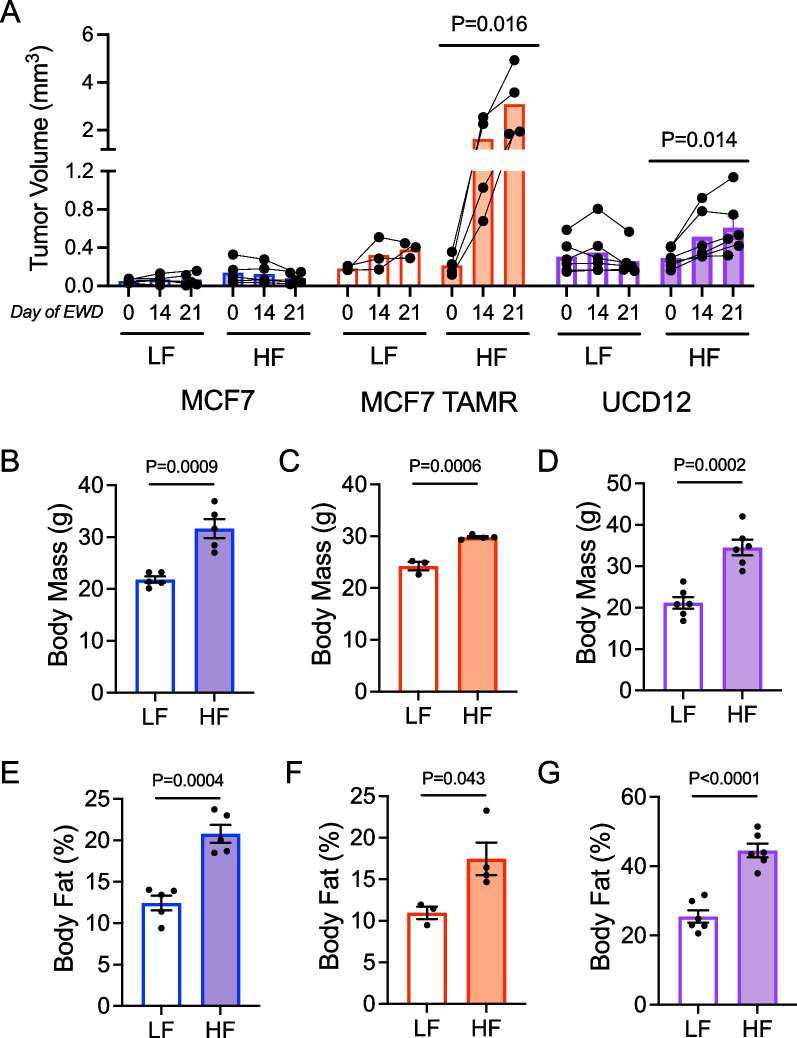
Estrogen receptor-positive tumor growth in low-fat- and high-fat-fed mice. **A** Volume of MCF7 (blue; *N* = 5 each), MCF7 TAMR (red; *N* = 3 LF, *N* = 4 HF), or UCD12 (purple; *N* = 6 each) tumors at days 0, 14, and 21 after estrogen withdrawal of ovariectomized female mice fed a low-fat or high-fat diet. Data analyzed by repeated measures ANOVA within groups. **B**–**D** Body mass at the end of study in mice bearing **B** MCF7 tumors, **C** MCF7 TAMR tumors, or **d** UCD12 tumors. **E**–**G** Body fat percentage at the end of study in mice bearing **E** MCF7 tumors, **F** MCF7 TAMR tumors, or **G** UCD12 tumors. Data were analyzed by unpaired *t*-tests

We used our established murine model of diet-induced obesity and ovariectomy to evaluate ER-positive tumor growth after estrogen deprivation [9, 16]. Consistent with our previous findings, MCF7 cell xenograft tumors did not grow in HFD-fed mice after ovariectomy and supplemental estrogen withdrawal (EWD; Fig. 1A). In contrast, tamoxifen-resistant MCF7 cells (MCF7 TAMR) and ER-positive UCD12 tumors grew significantly larger in HFD-fed (obese) females after EWD (Fig. 1A). In previously published studies, the UCD12 PDX did not regress significantly after either tamoxifen treatment or estrogen deprivation compared to estrogen supplementation when grown in chow-fed immune compromised mice [33]. This is consistent with what we observe in LFD-fed females here, and in previous work [9]. Body mass (Fig. 1B–D) and fat content (Fig. 1E–G) were greater in HFD- compared to LFD-fed females in all three studies. These experiments reiterate that not all ER-positive breast tumors continue to grow after simulated endocrine therapy in the context of obesity; however, the underlying reasons for these differences are unclear. Our prior studies implicated adipose-derived FGF1 in the obesity-driven growth of ER-positive breast tumors [9, 34]; therefore, we hypothesized that altered signaling downstream of FGF1 may influence the differential responses of tumors to estrogen deprivation in the context of obesity.

**Fig. 2:**
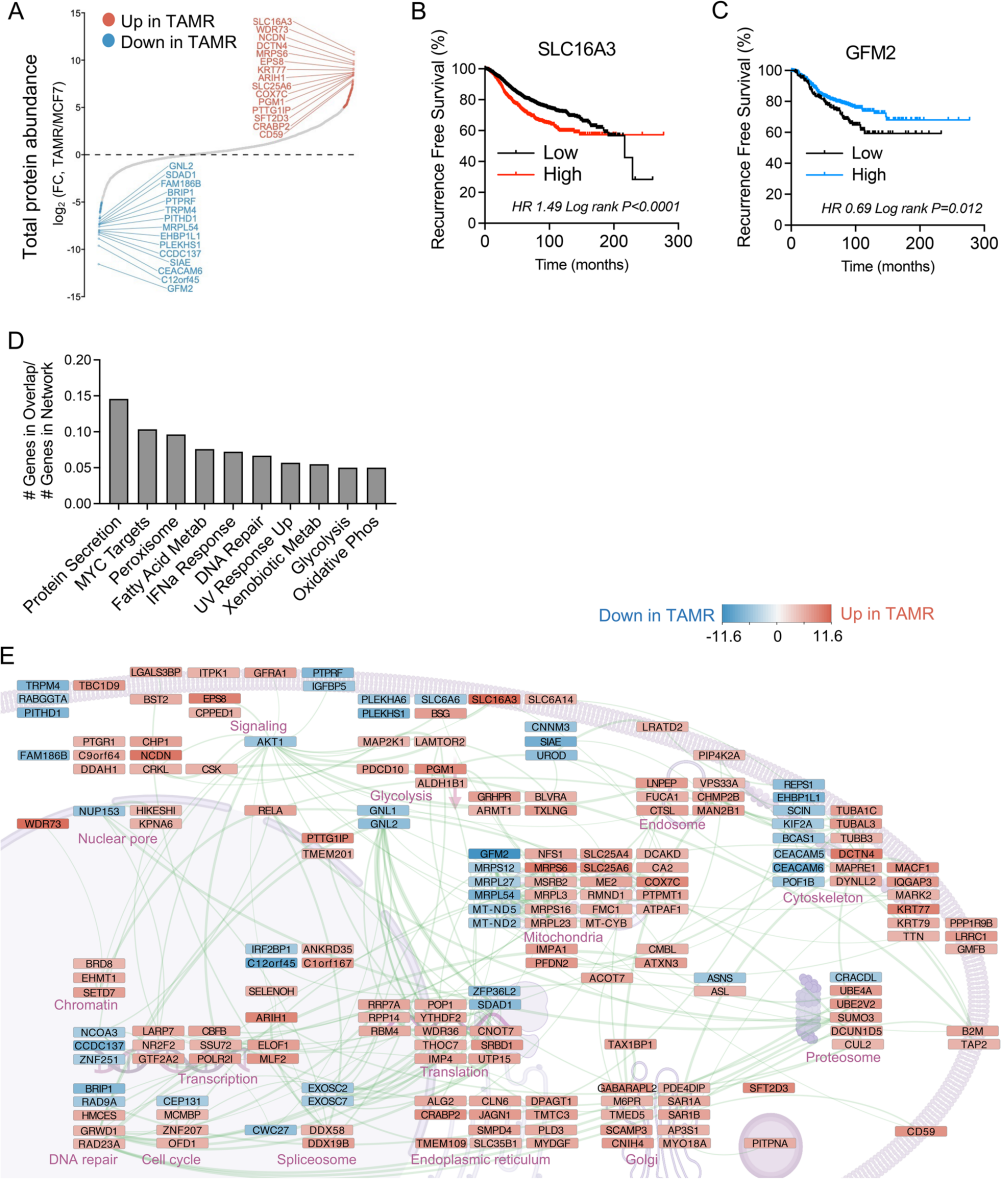
Untargeted proteomics analysis of MCF7 and MCF7 TAMR cells. **A** Total protein abundance measured by proteomics and expressed as the log2 fold change in MCF7 TAMR versus MCF7 cells. **B**–**C** Kaplan–Meier curves of recurrence-free survival percent for patients with ER-positive breast cancer based on high or low SLC16A3/MCT4 (**B**) or GFM2 (**C**). Data analyzed by log rank test. **D** Analysis of Hallmark pathways enriched in proteins elevated log2 fold change ≥ 2 in MCF7 TAMR versus MCF7. Data are expressed as k/K (number of genes in overlap/number of genes in network) and all were FDR *p*-value < 0.05. **E** Diagram of the cellular locations and functions of proteins enriched in MCF7 TAMR versus MCF7 cells. Red is enriched in MCF7 TAMR and blue is enriched in MCF7 (decreased in MCF7 TAMR). The color intensity of red or blue reflects the log2 fold change as shown in panel B. The connecting lines represent high-stringency protein–protein interactions identified using STRING network analysis

To better understand the distinct signaling pathways regulated by FGF1 and estradiol (E2) in these tumors, we conducted an unbiased proteomic exploration of MCF7 and MCF7 TAMR cell lines, which showed the most disparate responses to the obese environment. We detected 3610 unique proteins with high confidence. At baseline, we observed clear differences in protein abundance between these two cell lines (Fig. 2A; Additional file 2: Table S1). The most overrepresented protein in MCF7 TAMR cells was SLC16A3, which is a plasma membrane transporter for lactate, pyruvate, and ketone bodies, also known as MCT4 [35]. The MCT4 transporter is a key regulator of breast cancer cell metabolism [36, 37]. Expression of SLC16A3 in ER-positive breast tumors was associated with significantly shorter recurrence-free survival (RFS; Fig. 2B) as shown by others [38]. Conversely, the most underrepresented protein in MCF7 TAMR versus MCF7 cells was the mitochondrial translation elongation factor, GTP-dependent ribosome recycling factor mitochondrial 2 (GFM2; Fig. 1A), which plays a role in terminating mitochondrial translation. Little is known about this protein in the context of breast cancer, but genomic mutation and loss of GFM2 associates with impaired mitochondrial respiration in muscle [39]. In ER-positive breast tumors, high expression of the GFM2 gene is associated with longer RFS for patients (Fig. 2C). When we evaluated ER-negative tumors, SLC16A3 also predicted a shorter RFS (Additional file 1: Fig S1A), while GFM2 expression associated with a longer RFS, similar to the ER-positive subtype (Additional file 1: Fig S1B). Analyzing all tumor subtypes together suggested that the alterations in expression of glycolytic (SLC16A3)- and mitochondrial (GFM2)-associated genes may be a general feature of aggressive breast cancers (Additional file 1: Fig S1C, D).

Many other proteins were significantly different between the cell types, and a common theme was cellular metabolism. Among other altered proteins related to cellular metabolism were the mitochondrial ADP/ATP antiporter SLC25A6 (a.k.a. ANT3), the cytochrome C oxidase subunit COX7C (complex IV), and phosphoglucomutase 1 PGM1, indicating that MCF7 TAMR cells are likely to have altered mitochondrial and glycolytic metabolism that potentially contributes to how they respond to estrogen deprivation. Consistent with endocrine resistance, we identified key pathways that were represented by proteins elevated in MCF7 TAMR compared to parental MCF7 cells including protein secretion and DNA repair (Fig. 2D). Next, we generated high-stringency protein–protein interaction networks and mapped them onto their location/functions within the cell. Using this compartment-award network analysis approach, we observed upregulation of functional protein networks involved in proximal signaling (greater MAP2K1/MEK, LAMTOR2; lower AKT1), gene transcription, protein translation, endoplasmic reticulum, Golgi, and cytoskeleton (Fig. 2E). Together, these unbiased observations point to the potential for extensive metabolic rewiring in MCF7 TAMR cells, compared to the parental MCF7 line.

**Fig. 3:**
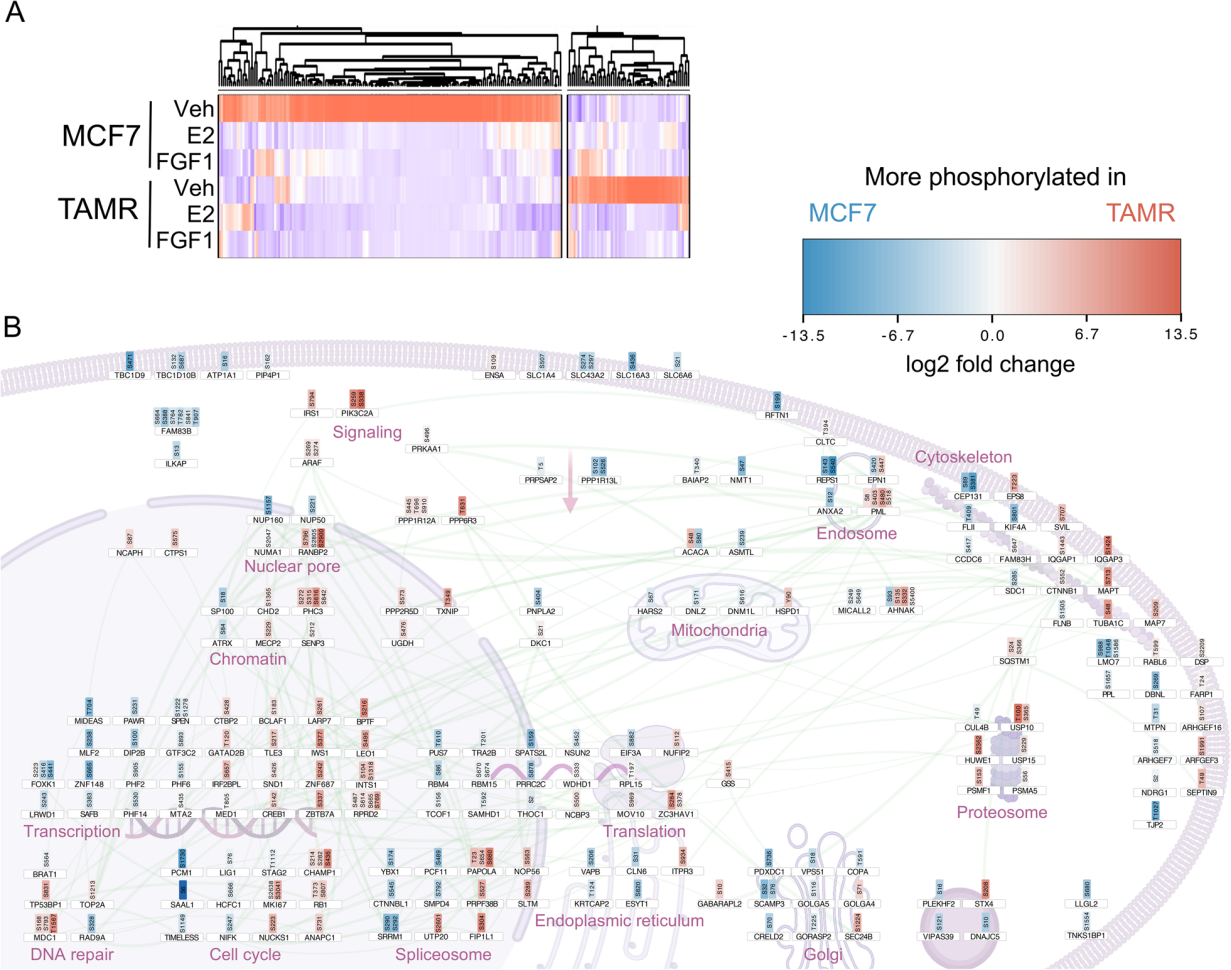
Steady-state phospho-proteomics analysis of ER-positive breast cancer cells. (A) Heatmap shows the hierarchical clustering of protein phospho-site abundance normalized to total protein data in MCF7 and MCF7 TAMR cells treated with Veh, E2 (10 nM), or FGF1 (5 ng/mL) for 15 min, emphasizing enriched phospho-proteins in vehicle-treated control conditions. (B) Diagram of residues phosphorylated in MCF7 TAMR versus MCF7 cells. Red sites are more phosphorylated in MCF7 TAMR and blue sites are more phosphorylated in MCF7 (decreased in MCF7 TAMR) in vehicle control conditions. The connecting lines represent high-stringency protein–protein interactions identified using STRING network analysis (see "Methods")

**Fig. 4:**
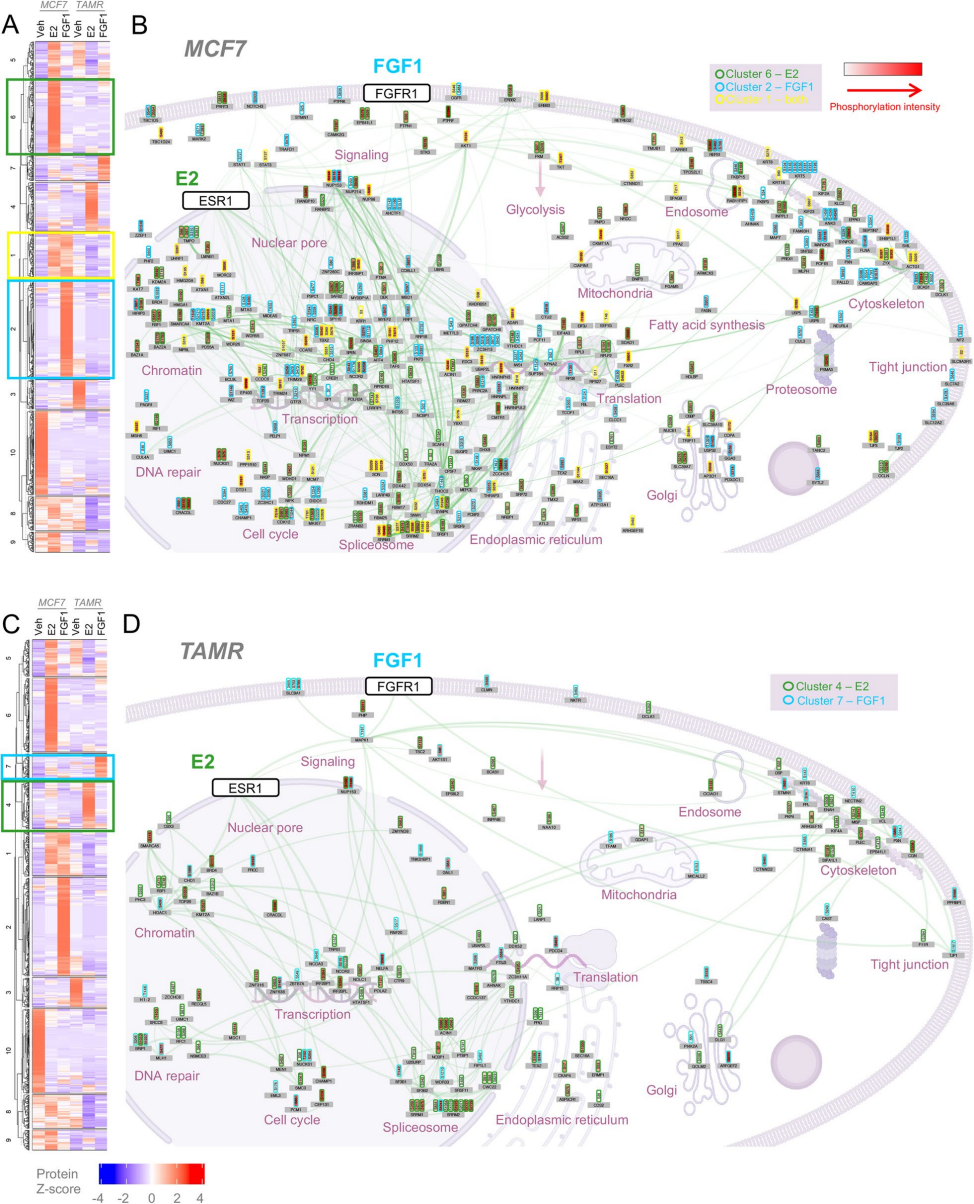
Phospho-proteomics analysis of E2- and FGF1-treated ER-positive breast cancer cells. **A** Heatmap shows the hierarchical clustering of protein phospho-site abundance normalized to total protein data in MCF7 and MCF7 TAMR cells treated with Veh, E2 (10 nM), or FGF1 (5 ng/mL) for 15 min, emphasizing enriched phospho-proteins in MCF7 cells. Clusters include proteins phosphorylated in response to E2 alone (cluster 6, green), FGF1 alone (cluster 2, blue), or common to both (cluster 1, yellow). **B** Diagram of residues phosphorylated in MCF7 cells that are unique to E2 (green outlines), FGF1 (blue outlines), or common to both (yellow outlines). **C** Heatmap of hierarchical clustering of protein phospho-site abundance normalized to total protein data in MCF7 and MCF7 TAMR cells treated with Veh, E2 (10 nM), or FGF1 (5 ng/mL) for 15 min, emphasizing enriched phospho-proteins in MCF7 TAMR cells. Clusters include proteins phosphorylated in response to E2 alone (cluster 4, green), FGF1 alone (cluster 7, blue). **D** Diagram of residues phosphorylated in MCF7 TAMR cells that are unique to E2 (green outlines) or FGF1 (blue outlines). No clusters were common to both treatments. For each diagram, red fill indicates phosphorylation intensity. The connecting lines represent high-stringency protein–protein interactions identified using STRING network analysis (see "Methods")

We also analyzed the phospho-proteome in MCF7 and MCF7 TAMR cells and detected a total of 4589 phospho-sites with high confidence. We observed distinct patterns of protein phosphorylation between cell lines (Fig. 3A; Additional file 3: Table S2). At steady-state, MCF7 TAMR cells exhibited elevated phosphorylation of proteins important for metabolic signaling (e.g., IRS1 S784, PIK3C2A S259, S338) and cell cycle (e.g., MKI67 S2638, S3041; RB1 T373, S807; Fig. 3B). E2 and FGF1 treatments elicited distinct responses in MCF7 and MCF7 TAMR cells (Fig. 4). Overall, there was evidence for a lower level of E2 or FGF1-dependent signaling above our significance threshold in MCF7 TAMR cells compared to MCF7 cells (Additional file 1: Fig S2, compare A,B to C,D). We first focused on MCF7 cells and compared the response to E2 (Fig. 4A, cluster 6) or FGF1 (Fig. 4A, cluster 2). The effects of each stimulus were widespread, and included proteins involved in transcription, splicing, translation, and cytoskeletal arrangement (Fig. 4B). Interestingly, there was a cluster of proteins phosphorylated by both E2 and FGF1 treatments (Fig. 4A, cluster 1) including ERBB3, STAT3, and AKT1 (Fig. 4B). In MCF7 cells, the most E2-responsive phospho-site was S1294 in protein tyrosine phosphatase receptor type F (PTPRF), a protein known to play a role in congenital breast hypoplasia [40] and murine mammary gland development [41] (Fig. 3B). On the other hand, one of the lowest abundance phospho-sites in this condition was S48 in acetyl Co-A carboxylase alpha (ACC1), the rate-limiting step in de novo lipogenesis.

In MCF7 TAMR cells, the response to either E2 or FGF1 treatments was not as robust as in the MCF7 cells and there were no proteins phosphorylated by both treatments at this time point (Fig. 4C, D). The scaffold attachment factor B2 (SAFB2), a repressor of ER, was phosphorylated at three sites by E2 in MCF7 cells, but not in MCF7 TAMR cells (Fig. 4B, D; Additional file 1: Fig S2). Pyruvate kinase M1/2 (PKM; S132, S249), phosphoglycerate mutase family member 5 (PGAM5 S80), human epidermal growth factor receptor 2 (ERRB2/HER2 S1083), and MKI67 T1503 were phosphorylated after E2 treatment in MCF7, but not MCF7 TAMR cells (Fig. 4B,D; Additional file 1: Fig S2). MCF7 TAMR cells gained the ability for E2 to phosphorylate the upstream mTOR regulator, TSC2, on S1132 (Fig. 4D). Also in these cells, FGF1 gained the ability to acutely activate MAPK1/ERK2 at Y187 (Fig. 4D), a key site for cell cycle regulation, the mTORC1 component AKT1S1/PRAS40 at S88, and that can phosphorylate ER independently of E2 stimulation. Interestingly, FGF1 treatment led to the robust phosphorylation of a co-activator of estrogen-responsive genes [42], protein glutamate and leucine-rich protein 1 (PELP1) on S658 in MCF7 cells, but this was not the case in MCF7 TAMR cells (Fig. 4B, D; Additional file 1: Fig S2). Collectively, these findings again suggest that signal transduction downstream of both E2 and FGF1 is re-wired in MCF7 TAMR cells, but in distinct ways.

### FGF1-induced ER phosphorylation in breast cancer cells

**Fig. 5:**
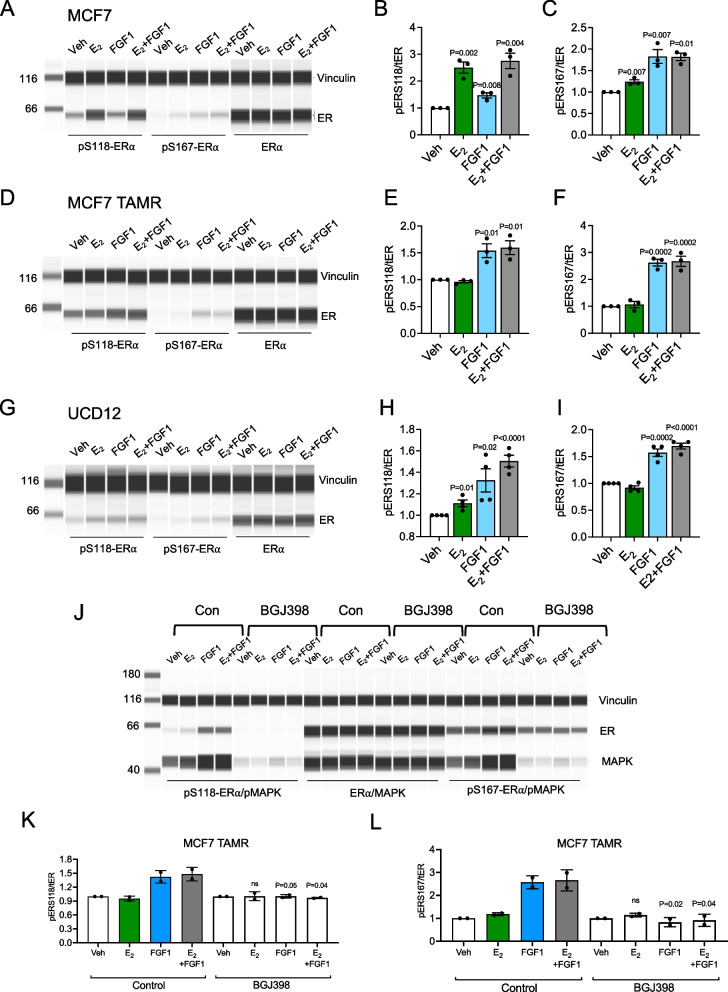
FGF1 can simulate ER phosphorylation in endocrine-resistant breast cancer cells. **A**–**C** Immunoblot analysis and quantification of pER-S118 (**B**), pER-S167 (**C**), and total ER in MCF7 cells after 15 min of treatment with vehicle (Veh), E2 (10 nM), FGF1 (5 ng/mL), or E2 + FGF1. **D**–**F** Immunoblot analysis and quantification of pER-S118 (**E**), pER-S167 (**F**), and total ER in MCF7 TAMR cells after 15 min of treatment with vehicle (Veh), E2 (10 nM), FGF1 (5 ng/mL), or E2 + FGF1. **G**–**I** Immunoblot analysis and quantification of pER-S118 (**H**), pER-S167 (**I**), and total ER in MCF7 cells after 15 min of treatment with vehicle (Veh), E2 (10 nM), FGF1 (5 ng/mL), or E2 + FGF1. All experiments were performed independently at least three times. Data were analyzed with unpaired *t*-tests, comparing each treatment to vehicle. **J** Full representative capillary immunoblot image of MCF7 TAMR cell lysates analyzed for vinculin (loading control), pER-S118, pER-S167, total ER, pMAPK, or total MAPK as indicated on the right, in cells treated with vehicle (Con) or BGJ398 (100 nM) overnight prior to stimulation with vehicle, E2, FGF1, or E2 + FGF1. **K**–**L** Quantification of immunoblot data in (**J**) from two independent experiments. Data are expressed as pER-S118/total ER (**K**) or pER-S167/total ER (**L**) and plotted as fold change of treatment versus vehicle. Data were analyzed by comparing the BGJ398 group to the control group within each treatment (vehicle, E2, FGF1, E2 + FGF1) using unpaired *t*-tests

**Fig. 6:**
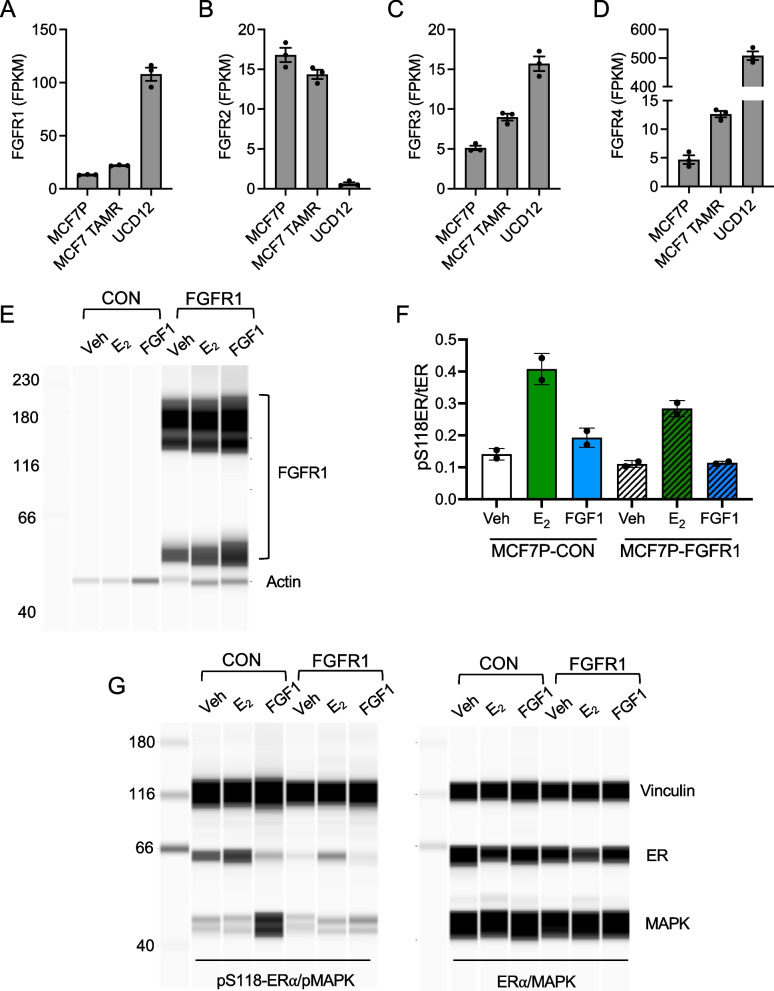
FGFR levels across cell lines and FGFR1 overexpression in MCF7 cells. **A**–**D** Expression levels of **A** FGFR1, **B** FGFR2, **C** FGFR3, and **D** FGFR4 in MCF7, MCF7 TAMR, and UCD12 cells measured by RNA sequencing. **E** Immunoblot analysis of FGFR1 and actin protein in control or FGFR1-overexpressing MCF7 cells treated with vehicle, E2, or FGF1 for 15 min. **F**–**G** Immunoblot analysis of pER-S118 and pER-S167 relative to total ER in control or FGFR1-overexpressing MCF7 cells treated with vehicle, E2, or FGF1 for 15 min. Representative immunoblots are shown in (**G**) with vinculin loading control, pMAPK, or total MAPK

FGFR signaling can activate ER without estrogen stimulation [43]; however, we were unable to measure ER phosphorylation with the untargeted proteomics approach. Using quantitative immunoblot, we evaluated ER phosphorylation at S118 and S167 in MCF7, MCF7 TAMR, and a third cell line derived from the PDX grown in LFD- and HFD-fed mice, UCD12 [17, 33] (Additional file 4: Table S3). Serines 118 and 167 are phosphorylated by MAPK and PI3K/AKT signaling, respectively, and are required for ER transcriptional activity [44, 45]. We previously showed that, as seen with the UCD12 PDX fragment, growth of the UCD12 cell line is sustained in HFD-fed mice after EWD [9]. In MCF7 cells, which do not continue to grow after EWD, E2 treatment led to greater S118 phosphorylation compared to FGF1 alone (Fig. 5A–B; Additional file 1: Fig. S3). E2 + FGF1 treatment resembled E2 (Fig. 5A–B). Phosphorylation of S167 was greater after FGF1 and E2 + FGF1 treatments compared to E2 alone (Fig. 5A, C). In contrast, treatment of MCF7 TAMR cells with FGF1 and E2 + FGF1 led to greater S118 phosphorylation than E2 alone (Fig. 5D–E). Phosphorylation of S167 in these cells was increased only by FGF1 or E2 + FGF1 (Fig. 5D, F). Similar to MCF7 TAMR cells, UCD12 cells showed greater S118 phosphorylation in response to FGF1 and E2 + FGF1 compared to E2 alone (Fig. 5G–H). Phosphorylation of S167 was similar between UCD12 and MCF7 cells, with greater levels seen after treatment with FGF1 or E2 + FGF1 compared to E2 alone (Fig. 5G, I). The effect of FGF1 on either ER phosphorylation site in MCF7 TAMR cells could be blocked with the FGFR inhibitor BGJ398 (Fig. 5J–L). While both serine residues were phosphorylated under some circumstances, it was the S118 site that showed a consistent relationship with the tumor growth phenotype in the obese environment (i.e., FGF1 associated with ER S118 phosphorylation in cells from tumors that grow without estrogen supplementation in obesity). In contrast, ER S167 phosphorylation was elevated with FGF1 treatment regardless of how the tumors grew in mice. Our previous study indicated that FGFR1 was overexpressed in UCD12 compared to MCF7 cells [9], and it was recently reported that FGFR1 was amplified in the UCD12 cell line [17]. Gene expression profiling of FGFR1-4 in all three cell lines showed different patterns of receptor expression across cells (Fig. 6A–D). To determine whether elevated FGFR1, as observed in UCD12 and MCF7 TAMR cells, was sufficient to allow FGF1-mediated ER phosphorylation at S118, we created MCF7-FGFR1-overexpressing cells and treated them with E2 or FGF1 (Fig. 6E). As expected in MCF7 control cells, E2 treatment increased ER phosphorylation at S118 and FGF1 did not (Fig. 6F-G). In MCF7-FGFR1 cells, E2 also increased ER phosphorylation, but FGF1 still did not at this time point (Fig. 6F-G), indicating that overexpression of FGFR1 may not be sufficient to promote FGF1-mediated ER activity during the same time course of treatment as the parental MCF7 cells. Overall, E2, FGF1, and the combination are capable of stimulating ER phosphorylation in each cell line, although with potentially different dynamics that may impact ER function and that are supported by widespread differences across cell lines in the total and phospho-proteome response to these stimuli.

**Fig. 7:**
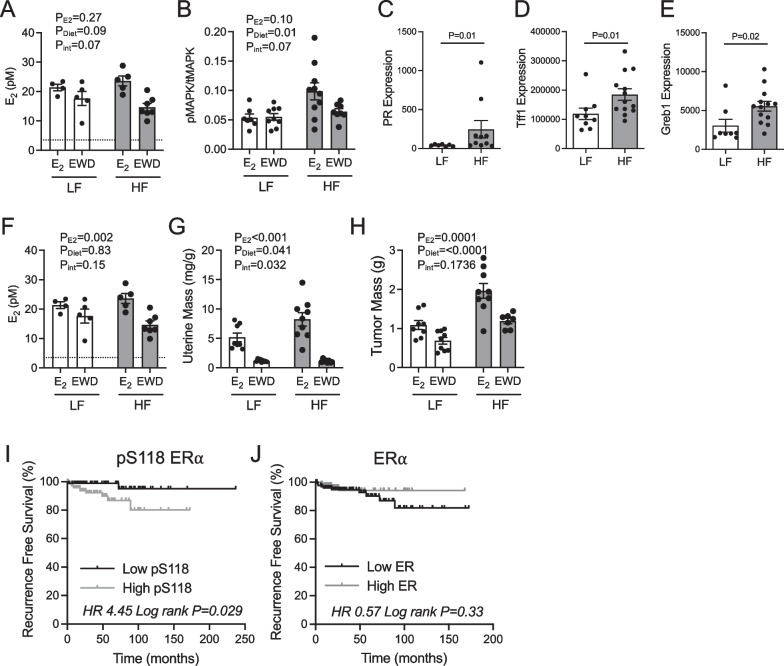
ER signaling in UCD12 tumors grown in low-fat- or high-fat-fed mice. **A**–**B** UCD12 tumor lysates from low-fat (LF)- or high-fat (HF)-fed mice treated with E2 or estrogen withdrawal (EWD) were used for immunoblot analysis of pER-S118 and total ER (**A**) or immunoblot analysis of pMAPK and total MAPK (**B**). **C**–**E** Expression of ER target genes progesterone receptor (PR; **C**), Tff1 (**D**), or Greb1 (**E**) was analyzed in UCD12 tumors from LF or HF E2-treated mice using qPCR. Data were analyzed using unpaired *t*-tests. **F** Estradiol measured in serum from fasted LF or HF E2- or EWD-treated ovariectomized mice. The dashed line indicates the lower limit of detection for the assay. **G** Uterine masses expressed as mg/g body mass in LF and HF E2- or EWD-treated ovariectomized mice. **H** Tumor masses at the end of study from LF and HF E2- or EWD-treated mice. **I**–**J** Kaplan–Meier plots of recurrence-free survival percent of patients with ER-positive breast tumors, with high or low levels of pER-S118 (**I**) or total ER (**J**), from the TCGA-RPPA dataset and include patients of any age, menopausal status, and treatment. Data were analyzed with log rank tests. Data in **A**, **B,** and **F**–**H** were analyzed using two-way ANOVA testing main effects of diet or E2 treatment with interaction

We next evaluated lysates from UCD12 tumors grown in LFD- and HFD-fed mice with and without E2 supplementation [9]. Both pS118 ER (Fig. 7A) and pMAPK (Fig. 7B) were elevated in E2-supplemented HF-fed females compared to all other groups (*p* = 0.07; Additional file 1: Fig S4). Tumor expression of ER target genes progesterone receptor (PR; Fig. 7C), growth-regulating estrogen receptor binding 1 (GREB1; Fig. 7D), and trefoil factor 1 (TFF1/pS2; Fig. 7E) were all significantly elevated in E2-treated HFD- versus LFD-fed females. Serum levels of E2 in mice were similar to those reported in postmenopausal women [46] and were not different between diet groups, regardless of supplementation (Fig. 7F); however, uterine masses clearly indicated the presence of estrogens in the E2-treated groups (Fig. 7G). Likewise, UCD12 tumors grown in E2-supplemented mice were larger than in mice who had undergone EWD (Fig. 7H). Notably, the assay used to measure serum E2 levels has a lower limit of detection of 3.5 pM, resulting in a potential overestimation of the levels present in serum after EWD. In lymph node-negative, ER-positive human breast tumors, high versus low levels of pS118 ER associated with a significantly poor outcome (Fig. 7I), but total ER protein levels did not (Fig. 7J). Together, these data suggest that ER may be activated independently of E2 in the context of obesity, which may impact tumor progression.

### Emergence of ER-dependent FGF1 signaling in endocrine-resistant breast cancer cells

**Fig. 8:**
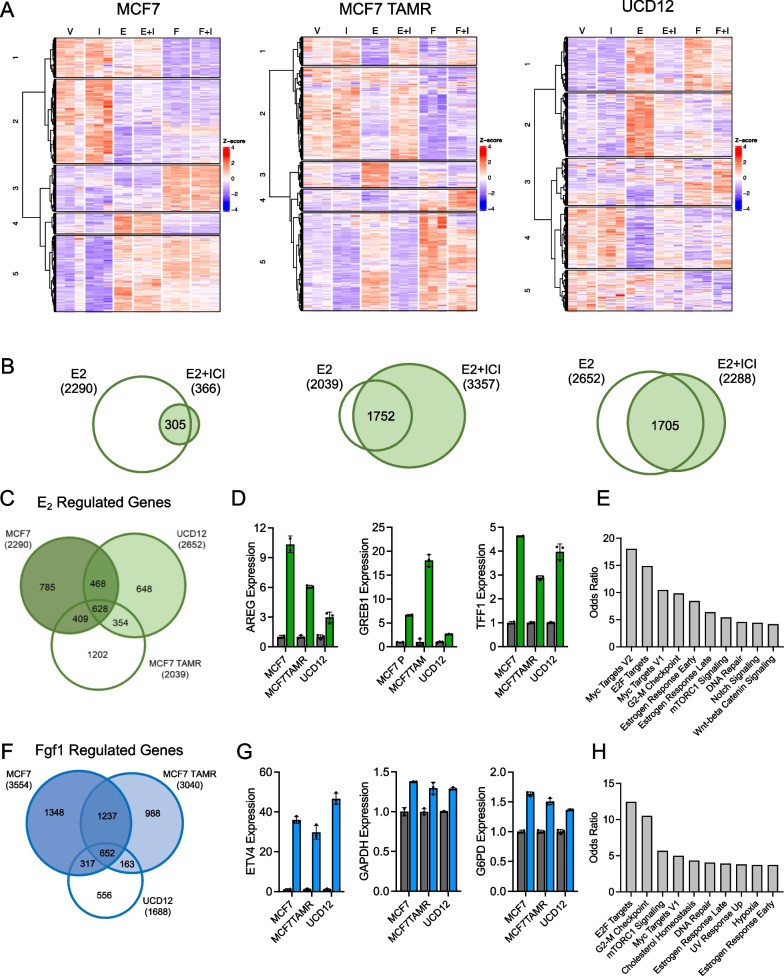
Gene expression profiling of E2 and FGF1-treated breast cancer cells. **A** Heatmaps of hierarchical clustering analysis of MCF7 (left), MCF7 TAMR (middle), and UCD12 (right) cells treated for 24 h with vehicle (V), fulvestrant/ICI (I), E2 (E), E2 + ICI (E + I), FGF1 (F), or FGF1 + ICI (F + I) and then analyzed with RNA sequencing. **B** Venn diagrams of the number of genes significantly (FDR *p* < 0.05) altered by E2 treatment or E2 + ICI treatment, and the overlap between those treatment groups. **C** Venn diagram of up- and down-regulated genes after 24 h of E2 treatment in MCF7, MCF7 TAMR, and UCD12 cells, all FDR *p* < 0.05. Green color represents E2 treatment. **D** Classical ER target genes Areg, Greb1, and Tff1 induced in all three cell lines. **E** Hallmark gene sets represented by genes that were commonly regulated by E2 in all three cell lines. **F** Venn diagram of up- and down-regulated genes after 24 h of FGF1 treatment in MCF7, MCF7 TAMR, and UCD12 cells, all FDR *p* < 0.05. Blue color represents FGF1 treatment. **G** Expression of glycolysis-associated genes Etv4, Gapdh, and G6PD in each cell line treated for 24 h with FGF1. **H** Hallmark gene sets represented by genes that were commonly regulated by FGF1 in all three cell lines

Based on the potential activation of ER and its target genes by FGF1, we performed RNA sequencing analysis of MCF7, UCD12, and MCF7 TAMR cells after 24 h of treatment with E2 or FGF1, or either one with the selective ER degrader fulvestrant (ICI) with the goal of identifying similarities and differences between the canonical ER ligand, E2, and non-canonical activation by FGF1 (Fig. 8A–B). Surprisingly, the relative impact of ICI on E2-regulated genes was greater in MCF7 TAMR and UCD12 cells than in MCF7 cells after 24 h of treatment (Fig. 8B). There was little overlap in E2-regulated genes among all three lines (Fig. 8C). Of the 628 that were common to all three, several classical ER target genes were present, including amphiregulin (AREG), GREB1, and TFF1 (Fig. 8D). Enriched Hallmark terms included estrogen response, Myc targets, and mTORC signaling (Fig. 8E). FGF1 targets were varied across cell lines as well (Fig. 8F). Enriched terms from genes that were greater or lower after FGF1 treatment are listed in Additional file 5: Table S4. In MCF7 cells, FGF1 increased expression of genes associated with hormonal response (Myc targets, estrogen response, androgen response) and also metabolic pathways. FGF1 treatment decreased genes associated with interferon response, estrogen response, and hypoxia. In MCF7 TAMR cells, FGF1 treatment increased metabolic genes and hypoxia targets, while in UCD12 cells, the uniquely upregulated FGF1 genes included those related to cell cycle progression and DNA repair. Conversely, FGF1 treatment decreased genes associated with estrogen signaling in MCF7 TAMR cells, and there were no significant pathways enriched in UCD12 cells from genes that were decreased by FGF1. Among the commonly regulated FGF1 genes was Ets variant transcription factor 4 (ETV4), which has been shown to influence ER DNA binding activity in endometrial cancer cells [47], and glycolytic gene expression in breast cancer cells [48] (Fig. 8G). Other FGF1-regulated genes included the glycolytic enzymes encoded by glyceraldehyde-3-phosphate dehydrogenase (GAPDH) and glucose-6-phosphate dehydrogenase (G6PD; Fig. 8G). The enriched terms in the FGF1 gene signature also included estrogen response targets (Fig. 8H).

**Fig. 9:**
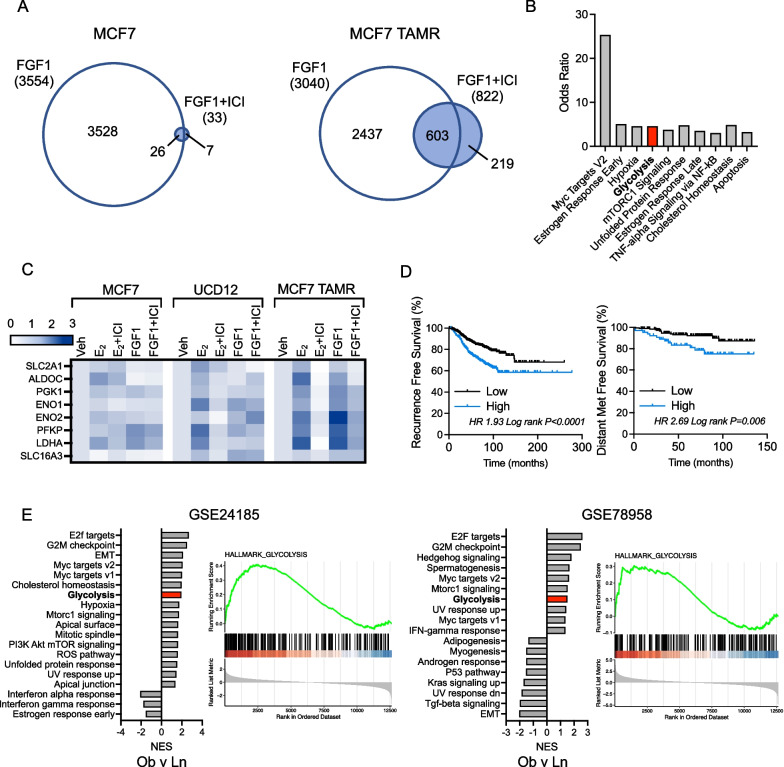
ER-dependent FGF1 signaling emerges with endocrine resistance. **A** Venn diagrams comparing the genes that were up- or down-regulated by FGF1, and reversed by co-treatment with fulvestrant (ICI) in MCF7 cells (left) or MCF7 TAMR cells (right), all FDR *p* < 0.05. **B** Hallmark gene set analysis of the 603 genes in MCF7 TAMR cells induced or repressed by FGF1 treatment and reversed by co-treatment with fulvestrant (ICI). Glycolysis is emphasized. **C** Heatmap of relative expression of 8 genes associated with glycolysis, expressed as fold change compared to vehicle (Veh) within each cell line. Genes include Slc2a1 (Glut1), Aldoc, Pgk1, Eno1, Eno2, Pfkp, Ldha, and Slc16a3 (MCT4). **D** Kaplan–Meier curves of recurrence-free survival % (left) and distant metastasis-free survival % (right) of patients with ER-positive breast cancer expressing high levels of glycolytic genes indicated in the heatmap in panel I. (K) GSEA analysis of dataset GSE24185 comparing ER-positive tumors from patients with obesity versus those without. Normalized enrichment scores (NES) of *p* < 0.05 gene sets are plotted. Glycolysis is emphasized in the bar graph (left) and in the GSEA plot (right). **E** GSEA analysis of datasets GSE24185 and GSE78958 comparing ER-positive tumors from patients with obesity versus those without. Normalized enrichment scores (NES) of adjusted *p* < 0.05 gene sets are plotted. Glycolysis is emphasized in the bar graphs and in the GSEA plots

We then compared the effect of fulvestrant (ICI) on FGF1-induced gene expression between MCF7 and MCF7 TAMR cells. There were very few FGF1-regulated genes whose expression was reversed by ICI in MCF7 cells (Fig. 9A). However, in MCF7 TAMR cells there were far more genes regulated by FGF1 that appeared to depend on ER (Fig. 9A). Among the enriched Hallmark terms of this gene set were hypoxia and glycolysis, which include many of the same genes (Fig. 9B). We more closely examined the expression of eight glycolytic genes that were induced by FGF1 and reversed by ICI and that contributed to the enrichment of the glycolysis signature across each cell line (Fig. 9C). In MCF7 cells, these genes showed no apparent regulation by E2 or FGF1, but in UCD12 cells, they appeared to be induced by E2 and reversed by ICI (Fig. 9C). In MCF7 TAMR cells, these genes showed a clear pattern of induction in response to E2 or FGF1, and the effect of each was reversed by ICI (Fig. 9C). The glycolytic gene expression profile is characteristic of aggressive triple-negative breast tumors that are associated with greater mortality than ER-positive tumors [49], but we found that high expression of these eight genes was also associated with significantly shorter recurrence- and distant metastasis-free survival in patients with ER-positive breast tumors, indicating a potentially relevant, aggressive subset of ER-positive breast cancer associated with some FGF1 target genes in endocrine-resistant breast cancer cells (Fig. 9D). Next, we evaluated enriched gene sets in ER-positive breast tumors from women with obesity compared to those without. Several gene sets that were positively enriched with obesity were also enriched in FGF1-treated MCF7 TAMR cells, including glycolysis (Fig. 9E), suggesting that obesity supports an environment of endocrine therapy resistance in ER-positive breast cancer, potentially prior to treatment. These observations are supported by extensive epidemiological data that show an adverse effect of obesity on the short- and long-term response of patients to endocrine therapy [50, 51]. Together, these data show that FGF1 signaling involves ER activation in endocrine-resistant obesity-associated breast cancers, and that one consequence may be metabolic reprogramming toward an aggressive glycolytic tumor phenotype.

### FGF1 alters glycolytic metabolism

**Fig. 10:**
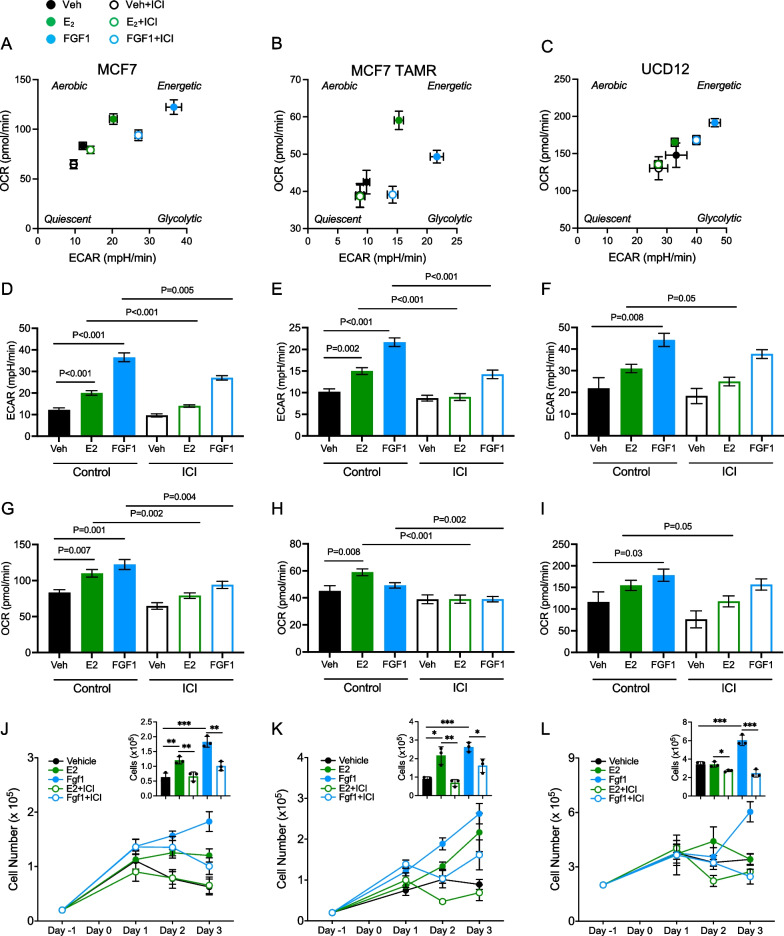
E2 and FGF1 treatments differentially influence cellular metabolism and proliferation of endocrine-sensitive and endocrine-resistant breast cancer cells. **A**–**C** Seahorse Analytics energy maps of MCF7 (**A**), MCF7 TAMR (**B**), and UCD12 (**C**) cells after 24 h of treatment with vehicle (filled black circle), E2 (filled green circle), FGF1 (filled blue circle), vehicle + ICI (open black circle), E2 + ICI (open green circle), or FGF1 + ICI (open blue circle). **D**–**F** Basal extracellular acidification rate (ECAR mpH/min) of MCF7 (**D**), MCF7 TAMR (**E**), and UCD12 (**F**) cells after 24 h of treatment with vehicle, E2, FGF1, or each with ICI. At least six wells per treatment are represented. **G**–**I** Basal oxygen consumption rate (OCR pmol/min) of MCF7 (**G**), MCF7 TAMR (**H**), and UCD12 (**I**) cells after 24 h of treatment with vehicle, E2, FGF1, or each with ICI. At least six wells per treatment are represented. **J**–**L** Cell number over 3 days of treatment with vehicle, E2, FGF1, E2 + ICI, or FGF1 + ICI for MCF7 (**J**), MCF7 TAMR (**K**), and UCD12 (**L**) cells. Insets show cell number at day 3 of the assay. For each graph, differences were estimated using *t*-tests

To understand how the changes in glycolytic gene expression affected cellular metabolism, we measured metabolic flux using the Seahorse XF Analyzer. In MCF7, MCF7 TAMR, and UCD12 cells, treatment with E2 or FGF1 shifted the overall metabolic phenotype toward glycolysis, compared to vehicle-treated cells (Fig. 10A–C). This was characterized by a significantly greater extracellular acidification rate (ECAR; a measure of glycolysis) after E2 or FGF1 treatments in all cell lines (Fig. 10D–F). Treatment with ICI lowered ECAR in E2- or FGF1-treated MCF7 and MCF7 TAMR cells (Fig. 10D–E) and slightly lowered ECAR in UCD12 cells treated with FGF1 (Fig. 10F). In MCF7 cells, the oxygen consumption rate (OCR; a measure of mitochondrial respiration) was significantly greater after E2 or FGF1 treatments (Fig. 10G); effects that were blocked by ICI. In MCF7 TAMR cells, OCR was greater after E2 treatment, but not after FGF1 treatment (Fig. 10H), and was lower after ICI treatment. UCD12 cells had elevated OCR after FGF1 treatment, and this was not blocked by ICI (Fig. 10I). We performed protein estimation assays after metabolic analysis to normalize the metabolic data; however, we saw distinct effects of treatments on protein levels across cell lines (Additional file 1: Fig. S5). In MCF7 cells, E2 and FGF1 treatment associated with greater protein content compared to vehicle control; effects that were reduced by fulvestrant (Additional file 1: Fig. S5A). In contrast, UCD12 and MCF7 TAMR cells showed no effect of E2, FGF1, or fulvestrant treatment on cellular protein content (Additional file 1: Fig. S5B-C). This could suggest that treatments with E2 or FGF1 only stimulated proliferation of MCF7, but not UCD12 or MCF7 TAMR cells, so we counted cells 1, 2, or 3 days after treatment. Cells in each line proliferated in response to both E2 and FGF1, with the greatest effect seen after FGF1 treatment (Fig. 10J–L). Notably, MCF7 and UCD12 cells treated with E2 appeared to plateau by day 2 (Fig. 10J, L), but in MCF7 TAMR, E2 treatment supported continued proliferation over the 3-day time course (Fig. 10K). Overall, E2 and FGF1 can alter cellular metabolism, potentially associated with cell proliferation but this may not be linked to protein production. The metabolic phenotype of FGF1-treated MCF7 TAMR cells, characterized by elevated ECAR and no change in OCR, is consistent with the glycolytic gene expression profile. The effects of E2 may be distinct in endocrine-sensitive (MCF7) versus endocrine-resistant (MCF7 TAMR) cells, whereas FGF1 is mitogenic regardless of the context. The effects of each treatment on cellular protein content depended on cell line, where we saw no association of protein levels with treatment in UCD12 or MCF7 TAMR cells. These results are consistent with protein translation and secretion pathway upregulation by E2 and FGF1 in the breast cancer cells that can progress in obese mice after EWD.

## Discussion

In this study, we identified potential mechanisms through which FGF1 supports ER-positive breast cancer growth after estrogen deprivation. First-line therapies for many breast cancer patients rely on blocking peripheral estrogen production with aromatase inhibitors, whether after menopause or in women receiving ovarian function suppression [52]. Our prior work indicated that, even without estrogen present, some ER-positive tumors continued to grow in the obese environment [9], and breast cancer endocrine therapy resistance is more frequent in patients with obesity [50, 51]. We surveyed the phospho- and total proteome of ER-positive breast cancer cell lines treated with E2 or FGF1. We also performed RNA sequencing analysis of cells to determine the differences between E2 and FGF1 target genes, particularly when ER was inhibited. We chose to further investigate FGF1 because of our previous work that showed elevated production during adipose tissue expansion and association with tumor FGFR activation and weight gain in HFD-fed females [9]. In this study, we found that, in cells from tumors that continue to grow after EWD in obese mice, E2 and FGF1 were capable of stimulating ER phosphorylation at S118 and S167, two key residues associated with ER activation. Together with the differences in total and phospho-proteins detected in untreated cells or with E2 or FGF1, our data suggest a complex alteration in protein signaling dynamics in ER-positive breast tumors that can grow without estrogen in obesity.

Various growth factors can act through ER and stimulate transcriptional activity even without estrogens. These include EGF, IGF1, and FGF2 and FGF7. FGFR signaling is known to associate with and promote endocrine therapy resistance in preclinical and clinical studies [15, 53]. Recently, FGFR1 overexpression was shown to promote proliferation, invasion, and cancer cell stemness in MCF7 and T47D breast cancer cells, associated with phosphorylation and activation of ER [54]. Mechanisms through which FGFR can activate steroid hormone receptors include activation of MAPK and PI3K pathways, which can then phosphorylate ER and the progesterone receptor (PR), as well as direct, nuclear interaction between FGFR and ER or PR that modulates target gene expression [11, 43, 55, 56]. MCF7 cells engineered to overexpress FGF1 achieved estrogen-independent growth in ovariectomized nude mice [57]. These tumors were highly vascularized and produced micro-metastases to the lung, but FGF1 overexpression was not sufficient to support the outgrowth of large metastatic lesions [57, 58]. FGF1 can be released from apoptotic or necrotic cancer cells into the tumor microenvironment where it can influence other cancer cells and can also be produced by surrounding adipocytes or stromal cells and contained in the extracellular matrix, as we have described [9]. In all three breast cancer cell lines examined, FGF1 treatment upregulated genes involved in cell proliferation (e.g., E2F targets, G2M checkpoint) and metabolism (e.g., cholesterol homeostasis, glycolysis), but there was not a significant enrichment of genes linked to invasion or metastasis.

We found that FGF1 can elicit phosphorylation and activity of ER in cells that display endocrine resistance in the obese environment (MCF7 TAMR and UCD12). Phosphorylation of ER and elevated levels of target genes were found in tumors from obese mice, supporting a role for ER in obesity-associated tumor progression, irrespective of E2 levels. Interestingly, functional evaluation of metabolic changes showed that FGF1 treatment enhanced glycolytic metabolism in all three cell lines, but in endocrine-resistant cells, the glycolytic phenotype was not accompanied by an increase in cellular oxygen consumption. Elevated glycolytic metabolism is a feature of many cancers and associates with aggressive cell behavior. The reasons for this are unclear, but may involve saturation or overload of mitochondrial metabolism [59]. The Seahorse experiments highlight a distinction between the effects of FGF1 on glycolysis (i.e., ECAR) and its effects on glycolytic gene expression. It is possible that a glycolytic gene signature in ER-positive tumors indicates a propensity for endocrine resistance that may be driven in part by FGF1. Whether or not this involves ER transcriptional activity remains to be determined. Together, these data indicate that FGF1 treatment can promote metabolic changes in cancer cells that may sustain their growth and highlight the potential to define FGF1-dependent gene expression signatures that predict endocrine resistance in obesity.

### Supplementary Information

**Additional file 1**.** Fig. S1** Analysis of human breast tumors. Kaplan–Meier curves of recurrence-free survival percent for all patients with breast cancer (A-B) or patients with ER-negative breast cancer (C-D) based on high or low SLC16A3/MCT4 (left) or GFM2 (right). Data analyzed by log rank test.** Fig. S2** Steady-state phospho-proteomics analysis of ER-positive breast cancer cells. (A) Heatmap shows the hierarchical clustering of protein phospho-site abundance normalized to total protein data in MCF7 and MCF7 TAMR cells treated with Veh, E2 (10nM), or FGF1 (5ng/mL) for 15 minutes, emphasizing enriched phospho-proteins in E2-treated conditions. (B) Diagram of residues phosphorylated in MCF7 TAMR (green) versus MCF7 (pink) cells. (C) Heatmap shows the hierarchical clustering of protein phospho-site abundance normalized to total protein data in MCF7 and MCF7 TAMR cells treated with Veh, E2 (10nM), or FGF1 (5ng/mL) for 15 minutes, emphasizing enriched phospho-proteins in FGF1-treated conditions. (D) Diagram of residues phosphorylated in MCF7 TAMR (green) versus MCF7 (pink) cells treated with FGF1. For both diagrams, red sites are more phosphorylated in MCF7 TAMR and blue sites are more phosphorylated in MCF7 (decreased in MCF7 TAMR) in vehicle control conditions. The connecting lines represent high-stringency protein–protein interactions identified using STRING network analysis (see "Methods").** Fig. S3** Analysis of MAPK and ER in breast cancer cells. Full representative capillary immunoblot images of multiplex evaluation of vinculin (loading control), pER-S118, pER-S167, total ER, pMAPK, or total MAPK in MCF7 cells, MCF7 TAMR cells, or UCD12 cells treated with vehicle (Veh), E2 (10 nM), FGF1 (5 ng/mL), or E2+FGF1 for 15 minutes following an overnight starve.** Fig. S4** Analysis of UCD12 tumors from LF- and HF-fed mice. (A) Light exposure capillary immunoblot images of pER-S118 along with pMAPK (top), or total ER along with total MAPK (bottom) in UCD12 tumor lysates from LF- or HF-fed mice treated with E2 or EWD. N=3 separate tumors per group. (B) Full representative capillary immunoblot image of UCD12 tumor lysates at a light exposure to show vinculin loading control.

**Additional file 2**. Table S1 Total protein abundance data for proteomics analysis on MCF7 and MCF7 TAMR cells.

**Additional file 3**. Table S2 Phospho-protein abundance data for proteomics analysis on MCF7 and MCF7 TAMR cells treated with vehicle, E2, or FGF1.

**Additional file 4**. Table S3 Analysis of immunoblot quantifications using one-way ANOVA with multiple comparisons.

**Additional file 5**. Table S4 Hallmark pathways enriched in gene expression changes in MCF7, MCF7 TAMR, and UCD12 cells treated with vehicle, E2, FGF1 or each with fulvestrant (ICI).

## Data Availability

Datasets are available as supplementary material or in the NCBI Gene Expression Omnibus.

## Abbreviations

BMI: Body mass index
FGF1: Fibroblast growth factor 1
FGFR1: Fibroblast growth factor receptor 1
ER: Estrogen receptor
E2: 17β-Estradiol
TAMR: Tamoxifen resistant
OCR: Oxygen consumption rate
ECAR: Extracellular acidification rate
