# Supplementary material for: FGF1 supports glycolytic metabolism through the estrogen receptor in endocrine-resistant and obesity-associated breast cancer
Source: Breast Cancer Res. 2023 Aug 22;25:99. doi: 10.1186/s13058-023-01699-0 (PMC10463730; doi:10.1186/s13058-023-01699-0)
Supplement: Supplementary file 1 — Additional file 1. Fig. S1 Analysis of human breast tumors. Kaplan–Meier curves of recurrence-free survival percent for all patients with breast cancer (A-B) or patients with ER-negative breast cancer (C-D) based on high or low SLC16A3/MCT4 (left) or GFM2 (right). Data analyzed by log rank test. Fig. S2 Steady-state phospho-proteomics analysis of ER-positive breast cancer cells. (A) Heatmap shows the hierarchical clustering of protein phospho-site abundance normalized to total protein data in MCF7 and MCF7 TAMR cells treated with Veh, E2 (10nM), or FGF1 (5ng/mL) for 15 minutes, emphasizing enriched phospho-proteins in E2-treated conditions. (B) Diagram of residues phosphorylated in MCF7 TAMR (green) versus MCF7 (pink) cells. (C) Heatmap shows the hierarchical clustering of protein phospho-site abundance normalized to total protein data in MCF7 and MCF7 TAMR cells treated with Veh, E2 (10nM), or FGF1 (5ng/mL) for 15 minutes, emphasizing enriched phospho-proteins in FGF1-treated conditions. (D) Diagram of residues phosphorylated in MCF7 TAMR (green) versus MCF7 (pink) cells treated with FGF1. For both diagrams, red sites are more phosphorylated in MCF7 TAMR and blue sites are more phosphorylated in MCF7 (decreased in MCF7 TAMR) in vehicle control conditions. The connecting lines represent high-stringency protein–protein interactions identified using STRING network analysis (see "Methods"). Fig. S3 Analysis of MAPK and ER in breast cancer cells. Full representative capillary immunoblot images of multiplex evaluation of vinculin (loading control), pER-S118, pER-S167, total ER, pMAPK, or total MAPK in MCF7 cells, MCF7 TAMR cells, or UCD12 cells treated with vehicle (Veh), E2 (10 nM), FGF1 (5 ng/mL), or E2+FGF1 for 15 minutes following an overnight starve. Fig. S4 Analysis of UCD12 tumors from LF- and HF-fed mice. (A) Light exposure capillary immunoblot images of pER-S118 along with pMAPK (top), or total ER along with total MAPK (bottom) in UCD12 tumor lysates from LF- or [file 13058_2023_1699_MOESM1_ESM.pdf]

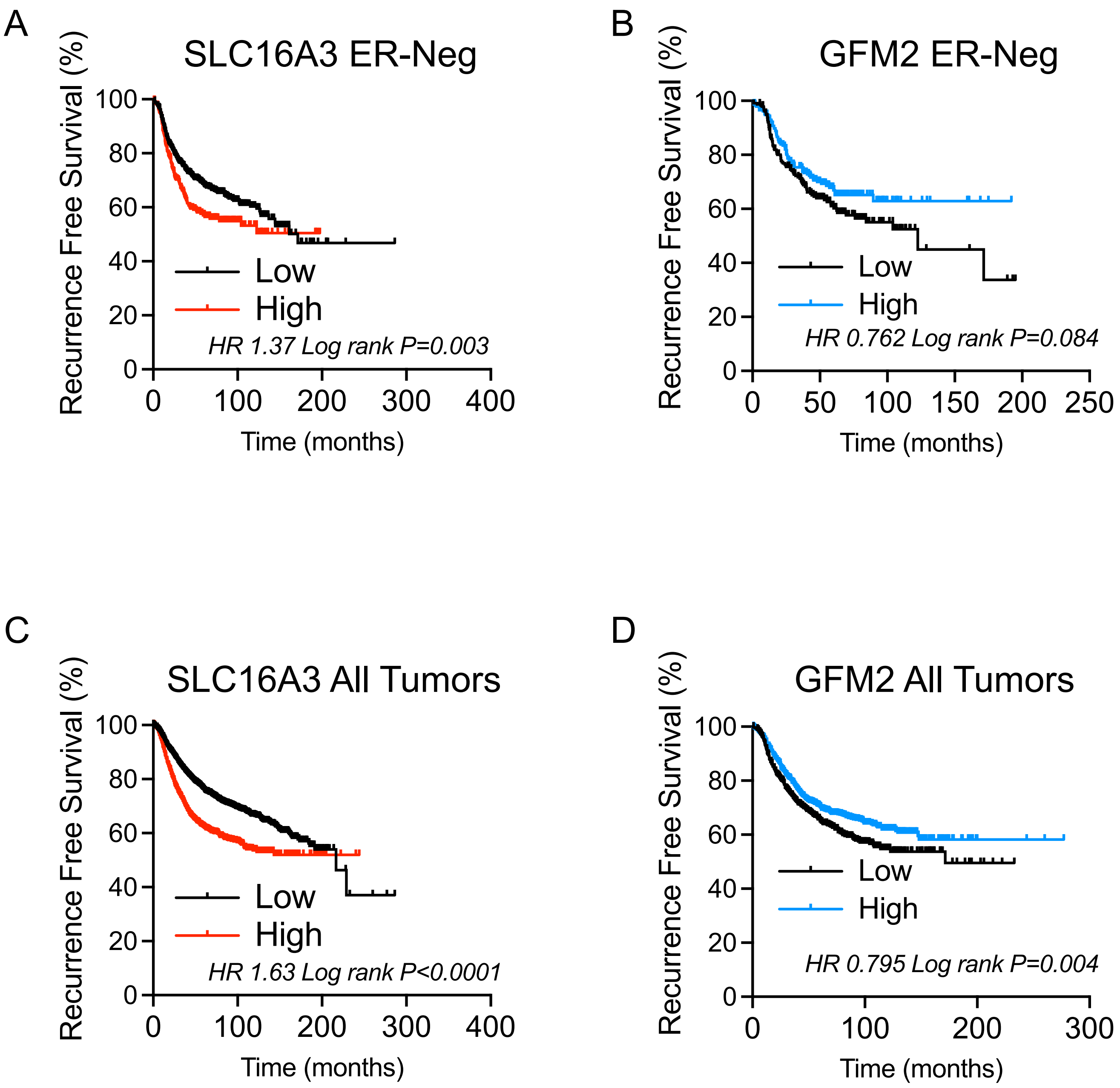

Supplemental Figure 1. Analysis of human breast tumors. Kaplan-Meier curves of recurrence free survival percent for all patients with breast cancer (A-B) or patients with ER-negative breast cancer (C-D) based on high or low SLC16A3/MCT4 (left) or GFM2 (right). Data analyzed by log rank test.

Supplemental Figure 2

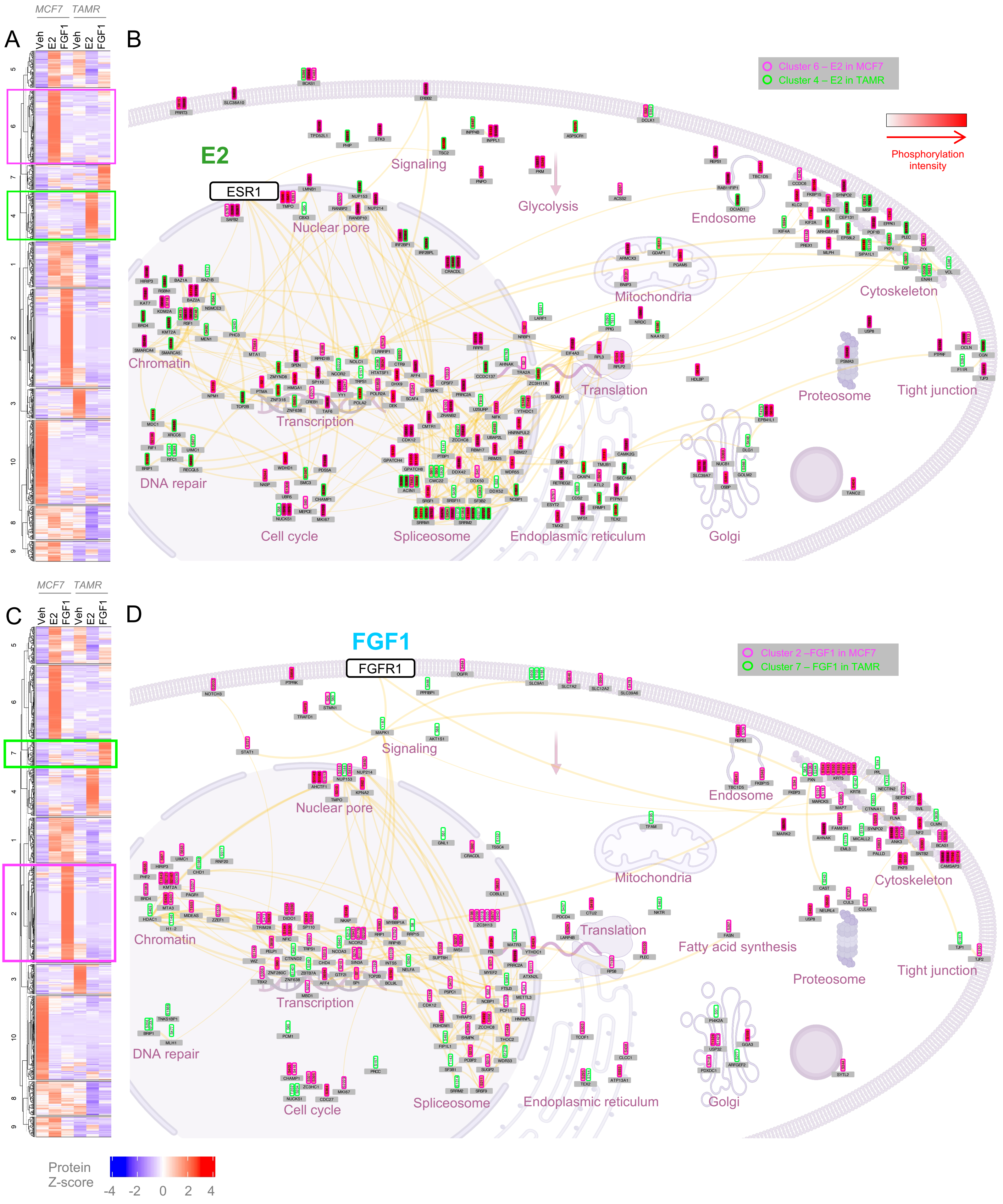

Supplemental Figure 2. Steady state phospho-proteomics analysis of ER-positive breast cancer cells. (A) Heatmap shows the hierarchical clustering of protein phospho-site abundance normalized to total protein data in MCF7 and MCF7 TAMR cells treated with Veh, E2 (10nM), or FGF1 (5ng/mL) for 15 minutes, emphasizing enriched phospho-proteins in E2-treated conditions. (B) Diagram of residues phosphorylated in MCF7 TAMR (green) versus MCF7 (pink) cells. (C) Heatmap shows the hierarchical clustering of protein phospho-site abundance normalized to total protein data in MCF7 and MCF7 TAMR cells treated with Veh, E2 (10nM), or FGF1 (5ng/mL) for 15 minutes, emphasizing enriched phospho-proteins in FGF1-treated conditions. (D) Diagram of residues phosphorylated in MCF7 TAMR (green) versus MCF7 (pink) cells treated with FGF1. For both diagrams, red sites are more phosphorylated in MCF7 TAMR and blue sites are more phosphorylated in MCF7 (decreased in MCF7 TAMR) in vehicle control conditions. The connecting lines represent high-stringency protein-protein interactions identified using STRING network analysis (see Methods).

MCF7

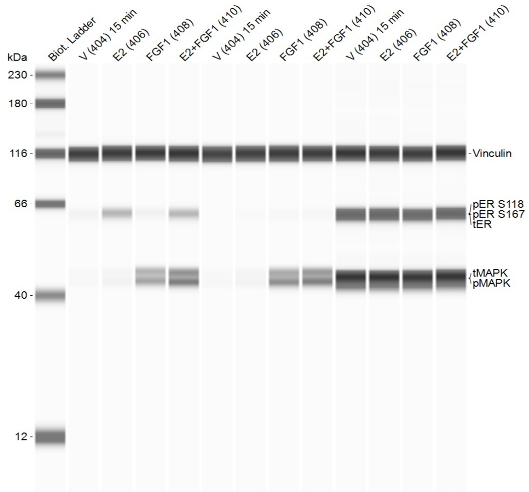

MCF7
TAMR

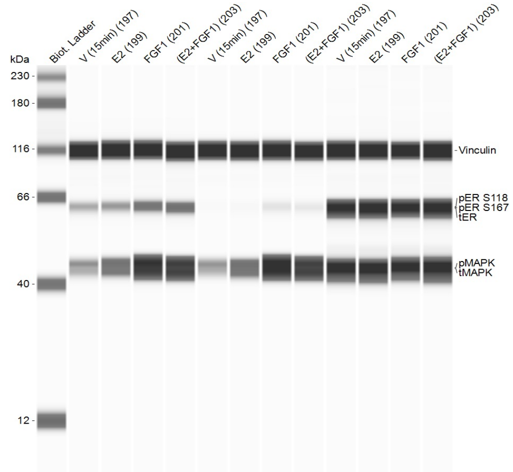

UCD12

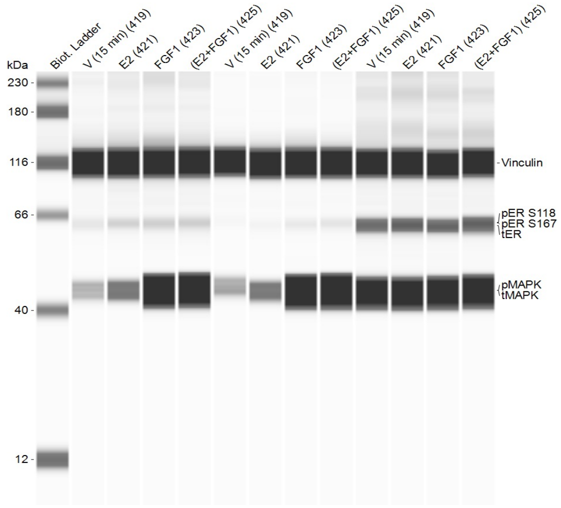

Supplemental Figure 3. Analysis of MAPK and ER in breast cancer cells. Full representative capillary immunoblot images of multi-plex evaluation of vinculin (loading control), pER-S118, pER-S167, total ER, pMAPK, or total MAPK in MCF7 cells, MCF7 TAMR cells, or UCD12 cells treated with vehicle (Veh), E2 (10 nM), FGF1 (5 ng/mL) or E2+FGF1 for 15 minutes following an overnight starve.

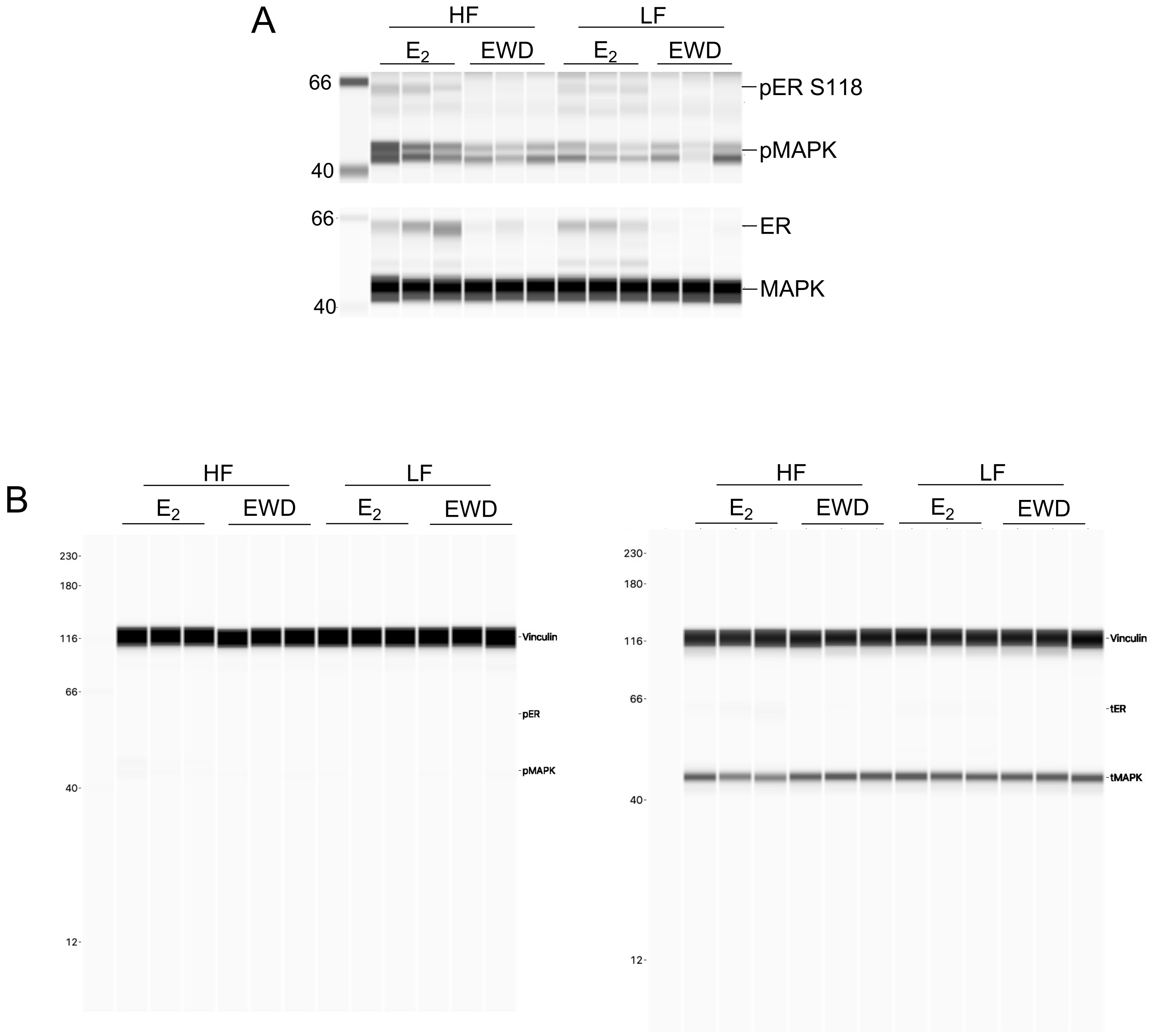

Supplemental Figure 4. Analysis of UCD12 tumors from LF and HF fed mice. (A) Light exposure capillary immunoblot images of pER-S118 along with pMAPK (top), or total ER along with total MAPK (bottom) in UCD12 tumor lysates from LF or HF fed mice treated with E2 or EWD. N=3 separate tumors per group. (B) Full representative capillary immunoblot image of UCD12 tumor lysates at a light exposure to show vinculin loading control.

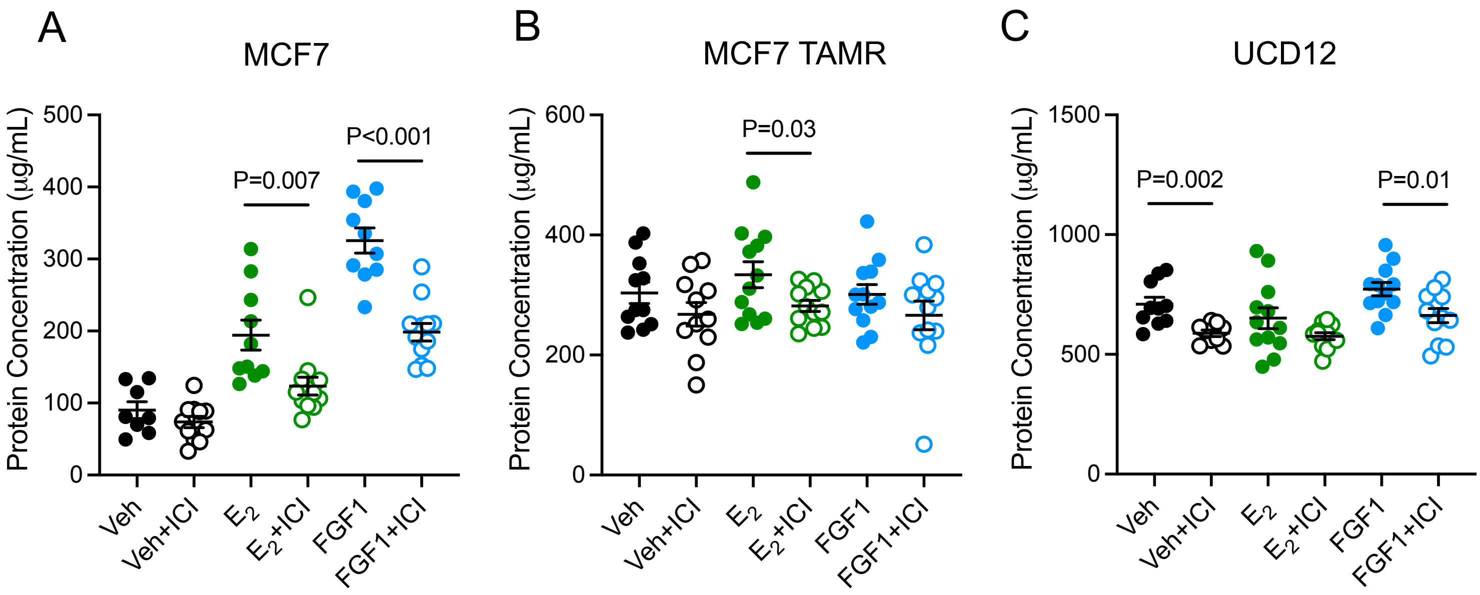

Supplemental Figure 5. Cellular protein content after Seahorse analyses. (A-C) BCA protein estimation assays after Seahorse metabolic analyses of MCF7 (a), MCF7 TAMR (b), or UCD12 (c) cells treated with vehicle, E2, FGF1, or each with ICI.
